# Data of methylome and transcriptome derived from human dilated cardiomyopathy

**DOI:** 10.1016/j.dib.2016.09.006

**Published:** 2016-09-14

**Authors:** Bong-Seok Jo, In-Uk Koh, Jae-Bum Bae, Ho-Yeong Yu, Eun-Seok Jeon, Hae-Young Lee, Jae-Joong Kim, Murim Choi, Sun Shim Choi

**Affiliations:** a Division of Biomedical Convergence, College of Biomedical Science, and Institute of Bioscience & Biotechnology, Kangwon National University, Chuncheon 24341, South Korea; b Division of Structural and Functional Genomics, Center of Genome Science, National Research Institute of Health, Chuncheongbuk-do 28159, South Korea; c Division of Cardiology, Cardiac and Vascular Center, Department of Medicine, Samsung Changwon Hospital, Sungkyunkwan University School of Medicine, Changwon 51353, South Korea; d Division of Cardiology, Seoul National University College of Medicine, Seoul 03080, South Korea; e Division of Cardiology, Asan Medical Center, University of Ulsan College of Medicine, Seoul 44033, South Korea; f Department of Biomedical Sciences, Seoul National University College of Medicine, Seoul 03080, South Korea

**Keywords:** Methylome, Transcriptome, DMP, DEG, DCM

## Abstract

Alterations in DNA methylation and gene expression have been implicated in the development of human dilated cardiomyopathy (DCM). Differentially methylated probes (DMPs) and differentially expressed genes (DEGs) were identified between the left ventricle (LV, a pathological locus for DCM) and the right ventricle (RV, a proxy for normal hearts). The data in this DiB are for supporting our report entitled “Methylome analysis reveals alterations in DNA methylation in the regulatory regions of left ventricle development genes in human dilated cardiomyopathy**”** (Bong-Seok Jo, In-Uk Koh, Jae-Bum Bae, Ho-Yeong Yu, Eun-Seok Jeon, Hae-Young Lee, Jae-Joong Kim, Murim Choi, Sun Shim Choi, 2016) [1].

**Specifications Table**

**Table:** 

Subject area	*Biology*
More specific subject area	*Epigenomics, Transcriptomics, Bioinformatics*
Type of data	*Tables and figures*
How data was acquired	*Infinium 450* *K HumanMethylation Bead chip and Human HT-12 v4 Expression BeadChip*
Data format	*Analyzed*
Experimental factors	*DMPs identified using RnBeads software. Statistical tests using R. And, a batch-scale comparison done by home-built Python script*
Experimental features	*Comparison of methylome and transcriptome between left ventricle (case) and right ventricle (control) in DCM patients*
Data source location	*National Institute of Health in Korea (KNIH)*
Data accessibility	*The data are within this article and deposited in GEO under accession number (GEO:*GSE81339*)*
	http://www.ncbi.nlm.nih.gov/geo/query/acc.cgi?token=erezeeaojpyvbqn&acc=GSE81339

**Value of the data**

• Provide a new insight on DNA methylation alteration in understanding the DCM etiology.
• Investigate the role of DNA methylation occurring in different genic regions associated with the regulation of gene expression.
• Provide new insights on the interaction network constructed by genes of DMP–DEG pairs.

## 1 Data

**Fig. 1:**
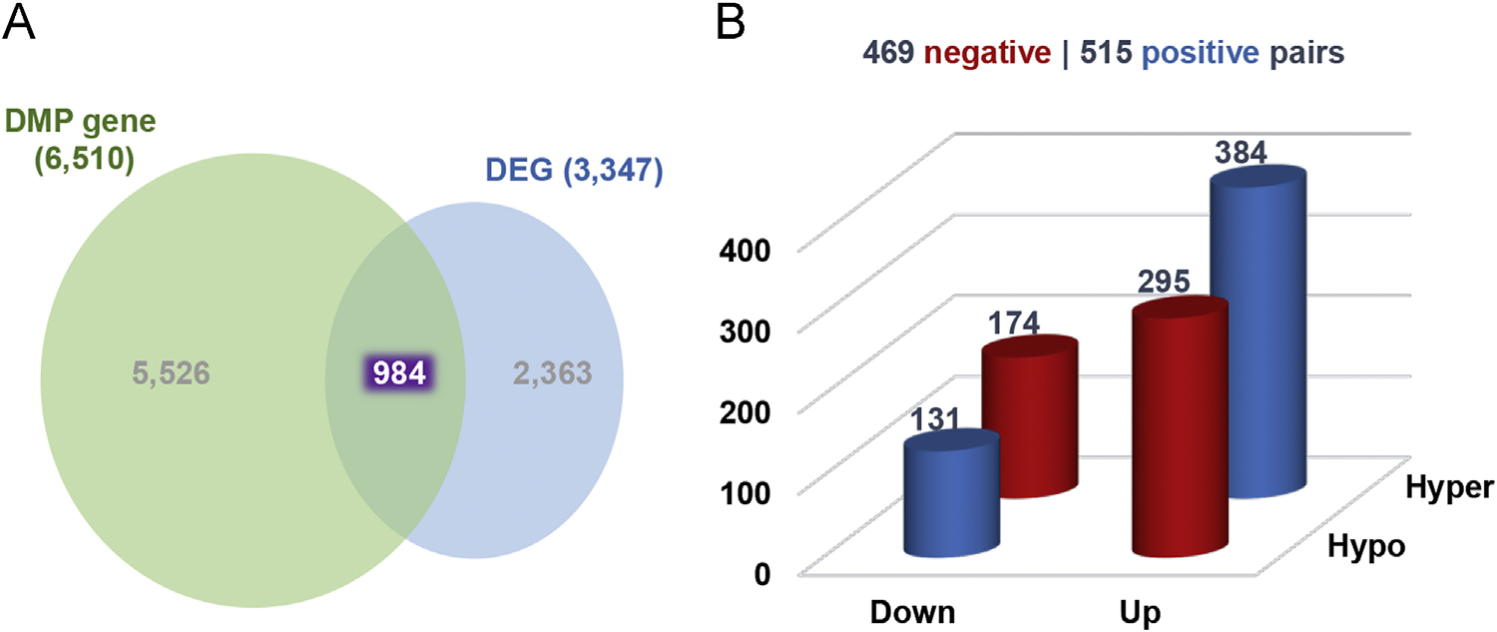
Analysis of the relationship between DNA methylation levels and gene expression. (A) A diagram showing the 984 overlapping genes between DMP-containing genes and DEGs. (B) Bar graphs showing the proportions of up- and down-regulated gene expression levels and up- and down-regulated DNA methylation levels. Red and blue indicate negative and positive relationships, respectively, between methylation levels and expression levels. ‘Up’ and ‘Down’: up- and down-regulated expression levels, respectively; ‘Hyper’ and ‘Hypo’: up- and down-regulated methylation levels, respectively.

**Fig. 2:**
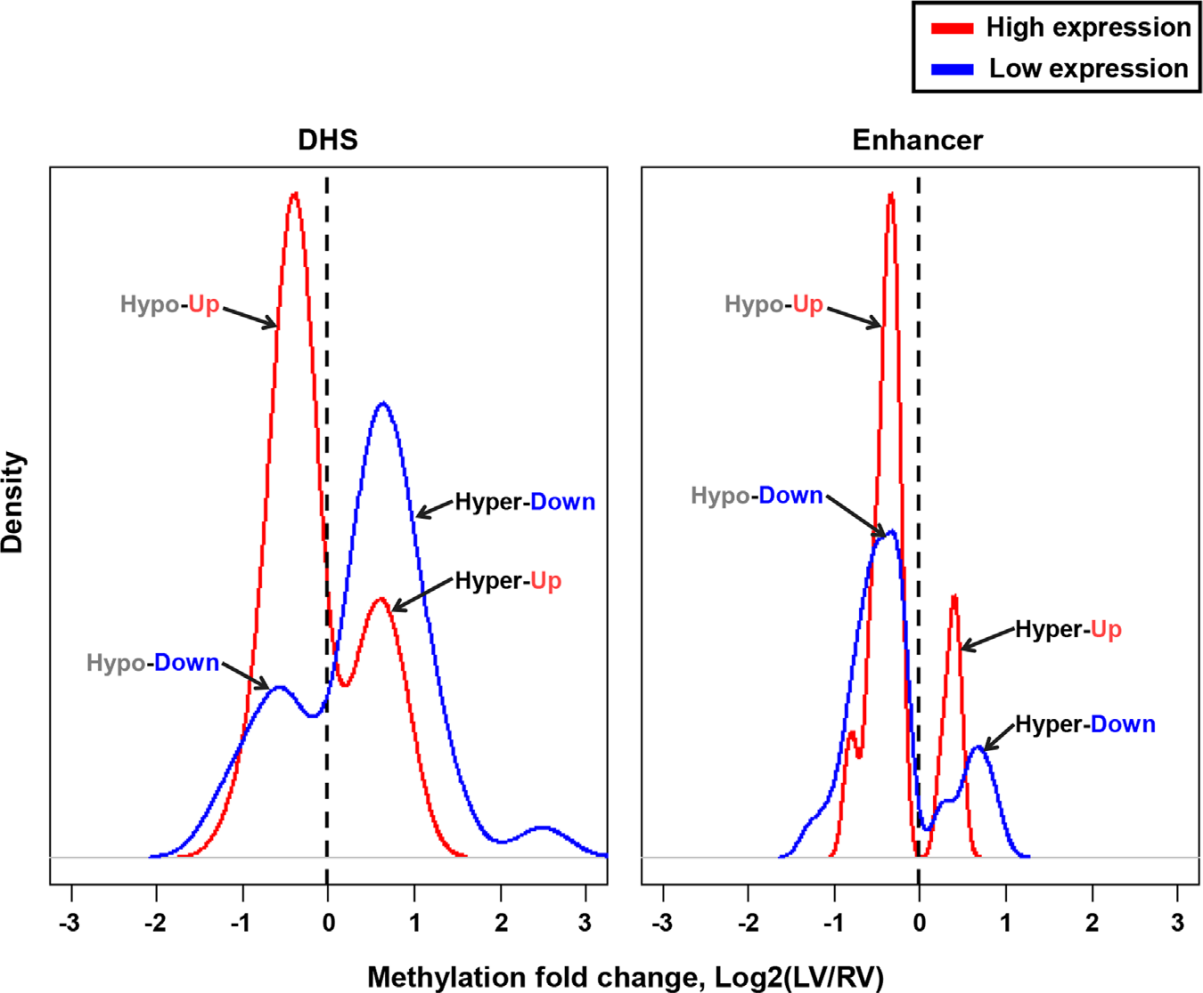
Methylation density of Up-/Down-DEGs in DHS and enhancer regions. Gene pairs with the top 20% of up-regulated expression fold-changes and bottom 20% of down-regulated expression fold-changes were selected among the 984 DMP–DEG pairs. The x-axis represent the fold-changes in methylation levels between RV and LV; negative values and positive values represent ‘Hypo’ and ‘Hyper’, respectively. The densities of ‘Hypo’ and ‘Hyper’ are plotted on the left (i.e., less-than-zero side) and right side (greater-than-zero side), respectively. The red lines represent the methylation densities of genes with the top 20% of up-regulated expression fold-changes (‘Up’), whereas the blue lines represent the methylation density of genes with the bottom 20% of down-regulated expression fold-changes (‘Down’).

**Fig. 3:**
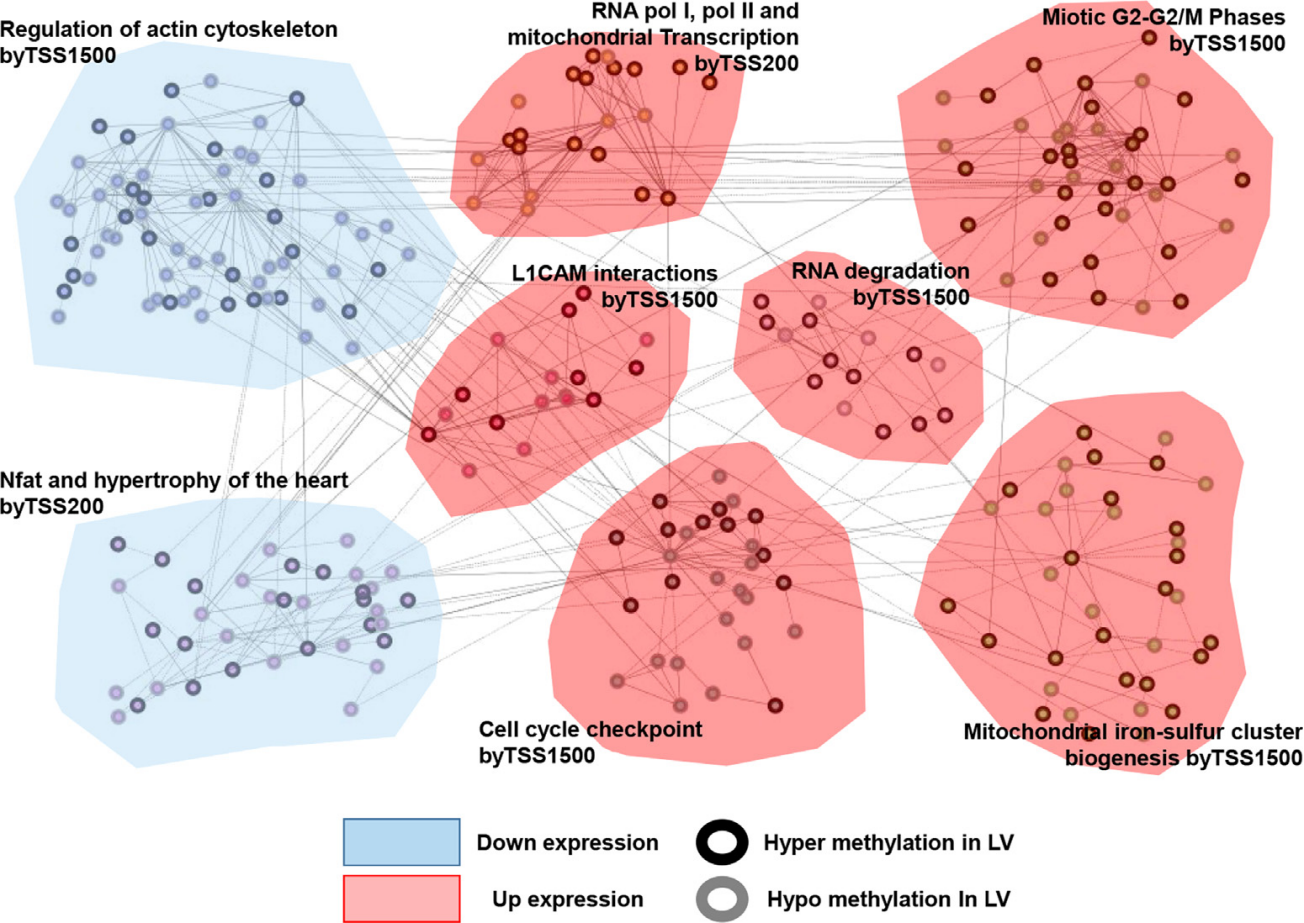
Functional network of DMP genes matched to DEGs by the Reactome pathway. Network lines represent an interaction in which mutual proteins are involved in the similar reaction. The eight top-ranked functional sub-networks with many nodes (genes) were selected from the 984 genes included in the input genes list. The most significant functional term in each sub-network was selected to indicate the pathway of each sub-network. The down- and up-regulation of expression in each sub-network was determined by the log2-transformed average fold change of the mean expression of the genes in a particular sub-network and colored blue and red, respectively. TSS1500 or TSS200 in a sub-network indicates that the DMP positions were relatively enriched in that sub-network. The bold black border of the nodes indicates hypermethylation in LV, whereas the gray border of nodes indicates hypomethylation in LV; the red area indicates grouped genes with up-regulated expression, whereas the blue area indicates grouped genes with down-regulated expression.

DCM samples where methylome and transcriptome data were produced used in the present DiB were listed in Table S1. The present data contain as followings: cleaning and normalization procedures (Figs. S1 and S2), global DNA methylation pattern (Figs. S3 and S4), multidimensional scaling (MDS) (Fig. S5), list of DMPs (Figs. S6 and S7; Table S2), identification of important variable probes (IVPs) (Figs. S8 and S9), DMP distribution in genic regions (Fig. S10), 984 DMP–DEG pairs (Fig. 1; Table S3), methylation alteration in DNase I hypersensitive site (DHS) and enhancer (Fig. 2), functional networks of the 984 DMP–DEG pairs (Fig. 3), gene ontology (Fig. S11), 45 cardiac ventricle development-related genes (Table 1), protein–protein interactions for the 45 genes (Fig. S12), and the relationship between methylation and expression of genes (i.e., TBX5 and HAND1) (Fig. S13).

## 2 Experimental design, materials and methods

### 2.1 Ethics statement

The data were prepared in accordance with principles (the Helsinki Declaration). It was approved by the Institutional Review Board (IRB) of The Samsung Medical Center (South Korea) (No. 2012-02-065). All participants have provided written informed consent and obtained the IRB approval for the consent procedure.

### 2.2 Tissue sample and chip data preparation from human DCM patients

Please refer to ‘Materials and Methods’ section of our original article published in Genomics [1] for the detailed procedures about where tissue samples originated from, how to extract DNAs and RNAs, and what kinds of chip technologies were used for data productions.

### 2.3 Finding DMPs and DEGs between LV and RV

One of the Bioconductor packages named RnBeads [2] was used for parsing raw intensity data generated from the Illumina 450 K IDAT files [3]. A total of 13,170 DMPs were chosen by a rank implemented by ‘combinedRank’ function of RnBeads program [2]. Please refer to our original paper [1] and the RnBeads program manual for the detailed protocols [2]. To identify DEGs, we first removed probes of detection *p*-value of over 0.01 in any sample and performed a quantile normalization [1]. Then, the filtered microarray data were compared between the LV and RV samples. A two-sample *t*-test was applied for selecting DEGs between the two samples at a FDR adjusted *p*<0.05 using R version 3.2.2 [4], from which a total of 3347 DEGs were identified.

### 2.4 Matching DMP–DEG pairs

Matching the 13,170 DMPs produced by the combinedRank cutoff (72,880) of RnBeads to the 3347 DEGs resulted in a total of 984 DMP–DEG pairs. This matching experiment was performed with home-built Python scripts.

### 2.5 Functional characterization of DMP-containing genes

Function of genes located at the nearest DMPs was estimated by a freely available web-tool called GREAT (http://bejerano.stanford.edu/great/public/html) [5]. Significance test for gene ontology (GO) enrichment was performed with the binomial test in the GREAT analysis. The protein–protein interaction network analysis for the selected 45 genes was performed with GeneMANIA (ver. 3.4.0) through the Cytoscape (ver. 3.2.0) [6].

## Figures and Tables

**Table 1:** List of the 45 genes characterized by GREAT.

**Gene Symbol**	**Official Full Name**
**ALX4**	ALX homeobox 4
**ARHGEF10**	Rho guanine nucleotide exchange factor (GEF) 10
**ATP2A1**	ATPase, Ca++ transporting, cardiac muscle, fast twitch 1
**BBC3**	BCL2 binding component 3
**BCL2**	B-cell CLL/lymphoma 2
**BRSK2**	BR serine/threonine kinase 2
**DNAJC10**	DnaJ (Hsp40) homolog, subfamily C, member 10
**EN1**	Engrailed homeobox 1
**FGF10**	Fibroblast growth factor 10
**FGF8**	Fibroblast growth factor 8 (androgen-induced)
**FOXC1**	Forkhead box C1
**FOXC2**	Forkhead box C2 (MFH-1, mesenchyme forkhead 1)
**FOXE3**	Forkhead box E3
**FOXF1**	Forkhead box F1
**GNB2L1**	Guanine nucleotide binding protein (G protein), beta polypeptide 2-like 1
**HAND1**	Heart and neural crest derivatives expressed 1
**HOXA3**	Homeobox A3
**HOXA5**	Homeobox A5
**HOXD11**	Homeobox D11
**ISL1**	ISL LIM homeobox 1
**ITPR1**	Inositol 1,4,5-trisphosphate receptor, type 1
**MECOM**	MDS1 and EVI1 complex locus
**MSX2**	msh homeobox 2
**MYBPC3**	Myosin binding protein C, cardiac
**MYL2**	Myosin, light chain 2, regulatory, cardiac, slow
**NKX2-5**	NK2 homeobox 5
**NOTCH1**	Notch 1
**OSR2**	Odd-skipped related transcription factor 2
**PPP1R13L**	Protein phosphatase 1, regulatory subunit 13 like
**PTCD2**	Pentatricopeptide repeat domain 2
**RAPGEF3**	Rap guanine nucleotide exchange factor (GEF) 3
**RBPJ**	Recombination signal binding protein for immunoglobulin kappa J region
**RDH10**	Retinol dehydrogenase 10 (all-trans)
**RYR2**	Ryanodine receptor 2 (cardiac)
**SIX1**	SIX homeobox 1
**SMAD3**	SMAD family member 3
**TBX5**	T-box 5
**TFAP2A**	Transcription factor AP-2 alpha (activating enhancer binding protein 2 alpha)
**TGFBR3**	Transforming growth factor, beta receptor III
**THRA**	Thyroid hormone receptor, alpha
**TMBIM6**	Transmembrane BAX inhibitor motif containing 6
**TNFRSF10B**	Tumor necrosis factor receptor superfamily, member 10b
**TNNC1**	Troponin C type 1 (slow)
**TWIST1**	Twist family bHLH transcription factor 1
**WNT7A**	Wingless-type MMTV integration site family, member 7A
