# Supplementary material for: Data of methylome and transcriptome derived from human dilated cardiomyopathy
Source: Data Brief. 2016 Sep 14;9:382–7. doi: 10.1016/j.dib.2016.09.006 (PMC5035344; doi:10.1016/j.dib.2016.09.006)
Supplement: Supplementary file 1 — Supplementary material [file mmc1.docx]

**Supplementary Table 1.** DCM samples used in DNA methylation profiling and mRNA microarray analyses.

| **Sample Name** | **Description detail** | **Sample group** | **Methylation**^a^ | **mRNA**  **expression**^b^ | **Sex** | **Age** |  |
| --- | --- | --- | --- | --- | --- | --- | --- |
| AMC01_LV2 | Cardiomyocyte, LV(apex) | case | o |  | M | 34 |  |
| AMC02_LV2 | Cardiomyocyte, LV (apex) | case | o | o | M | 50 |  |
| AMC03_LV2 | Cardiomyocyte, LV (apex) | case |  | o | F | 39 |  |
| AMC05_LV2 | Cardiomyocyte, LV (apex) | case |  | o | M | 65 |  |
| AMC06_LV2 | Cardiomyocyte, LV (apex) | case |  | o | F | 63 |  |
| AMC07_LV1 | Cardiomyocyte, LV | case |  | o | M | 63 |  |
| AMC07_LV2 | Cardiomyocyte, LV (apex) | case | o | o | M | 63 |  |
| AMC08_LV2 | Cardiomyocyte, LV (apex) | case |  | o | M | 31 |  |
| AMC09_LV1 | Cardiomyocyte, LV | case |  | o | M | 24 |  |
| AMC09_LV2 | Cardiomyocyte, LV (apex) | case |  | o | M | 24 |  |
| SMC01_LV2 | Cardiomyocyte, LV (apex) | case | o |  | F | 27 |  |
| SMC02_LV2 | Cardiomyocyte, LV (apex) | case | o |  | M | 41 |  |
| SMC03_LV2 | Cardiomyocyte, LV (apex) | case | o | o | F | 30 |  |
| SMC04_LV2 | Cardiomyocyte, LV (apex) | case | o |  | M | 61 |  |
| SMC05_LV2 | Cardiomyocyte, LV (apex) | case | o | o | M | 57 |  |
| SMC07_LV1 | Cardiomyocyte, LV | case |  | o | F | 43 |  |
| SMC07_LV2 | Cardiomyocyte, LV (apex) | case |  | o | F | 43 |  |
| SMC08_LV1 | Cardiomyocyte, LV | case |  | o | F | 72 |  |
| SMC08_LV2 | Cardiomyocyte, LV (apex) | case | o | o | F | 72 |  |
| SMC08_LV2-1 | Cardiomyocyte, LV (replication) | case | o |  | F | 72 |  |
| SNU01_LV2 | Cardiomyocyte, LV (apex) | case | o |  | M | 58 |  |
| SNU02_LV1 | Cardiomyocyte, LV | case |  | o | M | 92 |  |
| SNU02_LV2 | Cardiomyocyte, LV (apex) | case | o | o | M | 92 |  |
| SNU03_LV1 | Cardiomyocyte, LV | case |  | o | M | 45 |  |
| SNU03_LV1-1 | Cardiomyocyte, LV (replication) | case |  | o | M | 45 |  |
| SNU03_LV2 | Cardiomyocyte, LV (apex) | case | o | o | M | 45 |  |
| SNU04_LV2 | Cardiomyocyte, LV (apex) | case | o |  | M | 59 |  |
| SNU05_LV1 | Cardiomyocyte, LV | case |  | o | M | 52 |  |
| SNU05_LV2 | Cardiomyocyte, LV (apex) | case | o | o | M | 52 |  |
| AMC07_LV2_CF | Cardiac Fibroblast LV (apex) | case | o |  | M | 63 |  |
| SMC02_LV2_CF | Cardiac Fibroblast, LV (apex) | case | o |  | M | 41 |  |
| SMC08_LV2_CF | Cardiac Fibroblast, LV (apex) | case | o |  | F | 72 |  |
| AMC05_RV1 | Cardiomyocyte, RV | control | o |  | M | 65 |  |
| AMC06_RV1 | Cardiomyocyte, RV | control | o |  | F | 63 |  |
| AMC09_RV1 | Cardiomyocyte, RV | control | o | o | M | 24 |  |
| SMC01_RV1 | Cardiomyocyte, RV | control |  | o | F | 27 |  |
| SMC02_RV1 | Cardiomyocyte, RV | control |  | o | M | 41 |  |
| SMC07_RV1 | Cardiomyocyte, RV | control | o | o | F | 43 |  |
| SMC08_RV1 | Cardiomyocyte, RV | control | o | o | F | 72 |  |
| SNU02_RV1 | Cardiomyocyte, RV | control | o | o | M | 92 |  |
| SNU03_RV1 | Cardiomyocyte, RV | control | o | o | M | 45 |  |
| SNU04_RV1 | Cardiomyocyte, RV | control | o | o | M | 59 |  |
| SNU05_RV1 | Cardiomyocyte, RV | control | o |  | M | 52 |  |
| LV, Left ventricle. RV, Right ventricle. M, male. F, female.  ^a^Illumina infinium 450K Beadchip and ^b^HumanHT-12 v4 Expression BeadChip was used for methylation and mRNA expression, respectively.  AMC, SMC and SNU represented for a hospital name that each sample was obtained. | | | | | | | |
