# Supplementary material for: Data of methylome and transcriptome derived from human dilated cardiomyopathy
Source: Data Brief. 2016 Sep 14;9:382–7. doi: 10.1016/j.dib.2016.09.006 (PMC5035344; doi:10.1016/j.dib.2016.09.006)
Supplement: Supplementary file 5 — Supplementary material [file mmc5.pdf]

SUPPLEMENTARY FIGURES

Supplementary Figure 1:
Adjustment of color bias between the 450K Infinium I and Infinium II assays
Color bias adjustment was performed between Infinium I and Infinium II using the lumi package in R.

Supplementary Figure 2:
Data normalization
A normalization process was performed to remove non-biological noise caused by batch effect and probe type bias. The red and green bars represent the red and green channels of the chip, respectively, and the values along the y-axis represent the log2-converted intensity ratios of methylated and unmethylated probes. The BMIQ normalization method was applied to correct the probe bias generated by different types of probes.

Supplementary Figure 3:
Global DNA methylation patterns by CGI, promoter and gene body
(A) The mean β-values of CGIs, N/S shore, and N/S shelf were calculated for all probes of LV and RV after filtering and normalization. Island, CpG island; Shore, up to 2 kb from an island; Shelf, 2 kb to 4 kb from a CGI; Open sea, genomic CpGs not contained in the island, shore, and shelf. **(B)** The proportional distribution of CpG probes was plotted for each promoter and gene body region. The relative coordinates of 0 and 1 correspond to the start and end coordinates of a region, respectively. Coordinates less than 0 and greater than 1 denote the flanking regions of a region normalized by the region length.

Supplementary Figure 4:
Global DNA methylation patterns by genic regions
(A) The mean β-values for all CpG sites were plotted across all the ranges of β-values between 0 and 1. **(B)** Methylation of genomic regions was calculated as the mean β-value for all probes located within the region as annotated by the '450K chip', which covers the TSS1500, TSS200, 5'UTR, first exon, gene body, and 3'UTR. Error bars represent the standard error of the mean (SEM).

Supplementary Figure 5:
MDS plot analysis of DNA methylation between LVs and RVs
Red dots and cyan dots represent the 18 LVs and 9 RVs, respectively. MDS was performed with β-values estimated with CpG probes in the 450K chip separately for promoters, CGIs, and all CpG sites.

Supplementary Figure 6:
Volcano plot of β-value difference versus –log10 (FDR-corrected p-value)
The red dots represent the 1,828 significant DMPs where the methylation difference between the LV and RV was at a β-value > 5%, with an FDR-corrected p ＜ 5.0e-04. Gray dots represent all probes identified as DMPs by automatically generated by the 72,880 threshold of combinedRank by RnBeads.

Supplementary Figure 7:
Methylation similarity between our affected RV samples and normal, unaffected heart samples
(A) A heat map analysis combined with hierarchical clustering of the 1,828 DMPs was performed for our DCM samples and normal, unaffected heart samples (2RVs and 1LV, n = 3) downloaded from GEO (GSE16256). (**B)** Complete linkage hierarchical clustering was performed on relative methylation values (z-scores). LV and RV clusters represent our DCM samples, and N groups represent the normal unaffected samples. Note that the N samples cluster more closely to the RV samples than to the LV samples.

Supplementary Figure 8:
Schematic of the random forest analysis for identifying important variables that classify the LV and RV
To identify important DCM classifiers, a random forest algorithm was applied to our samples. One of R packages named 'randomForest' was used for this analysis. Important parameters for running the algorithm are as follows: 'ntree', the number for determining how many times decision trees are randomly created, '10,000' was used as input for our experiment; 'mtry', the number of input variables randomly chosen, 1,828 was used as the input in the present work. 'Out-of-bag estimate of error rate' is for error estimation. The 'MeanDecreaseGini' score is for measuring the variable importance used for the calculation of splits during training.

Supplementary Figure 9:
Validating 1,828 DMPs as a significant classifier
The 7,000 IVPs selected by the 'random forest' algorithm depicted in Fig. S8 were matched to 1,828 DMPs and 1,828 randomly chosen probes during 1,000 iterations. The bar in 'Random' represents the average of fractions of IVPs estimated for 1,828 randomly chosen probes during the iterating experiments. The error bar indicates the SEM. The bar in '1,828 DMPs' indicates the fraction of IVPs mapped to the real 1,828 DMPs.

Supplementary Figure 10:
Distribution of 13,170 DMPs selected by combinedRank threshold
The 13,170 DMPs were divided into two groups: hypermethylated DMPs (‘Hyper’ sites) and hypomethylated DMPs (‘Hypo’ sites). Distributions of the 13,170 DMPs were investigated throughout each genic region. Proportions of ‘All sites (all methylated sites)’, ‘Hyper’ sites, and ‘Hypo’ sites were calculated by genic locations divided into TSS1500, TSS200, 5’UTR, first exon, gene body and 3’UTR. Subsequently, proportions of hyper- and hypomethylated sites were compared to the proportion of all methylated sites detected by the 450k chip. An asterisk denotes a statistical significance at a $p < 0.05$ by the chi-squared test for comparisons.

Supplementary Figure 11:
Non-coding cis-regulatory regions of 45 cardiac ventricle development-related genes
(A) Graphic summaries were obtained by entering the chromosomal position of the 1,828 DMPs into the search box of the web-based GREAT tool (http://bejerano.stanford.edu/great/public/html/). **(B), (C)** Using the same GREAT website, mouse phenotypes and expression ontology provided by Mouse Genomic Informatics (MGI) were searched for the 1,828 DMPs.

Supplementary Figure 12:
Protein-protein interaction network constructed for the 45 genes
The 45 selected genes were used as inputs for GeneMANIA (a Cytoscape plug-in tool) by setting the physical interaction and consolidated pathway as the interaction parameters (see Materials and Methods).

Supplementary Figure 13:
Example views of DEGs that are negatively regulated by differential DNA methylation
(A) Significantly hypermethylated non-coding cis-regulatory regions of FGF8 and DNAJC10 are coupled with their down-regulated expression in LV. **(B)** Significantly hypomethylated non-coding cis-regulatory regions of HAND1 and TBX5 are coupled with their up-regulated expression in LV. FC: fold change of expression. The significance of these analyses was verified by Student’s t-test ($p < 0.01$).

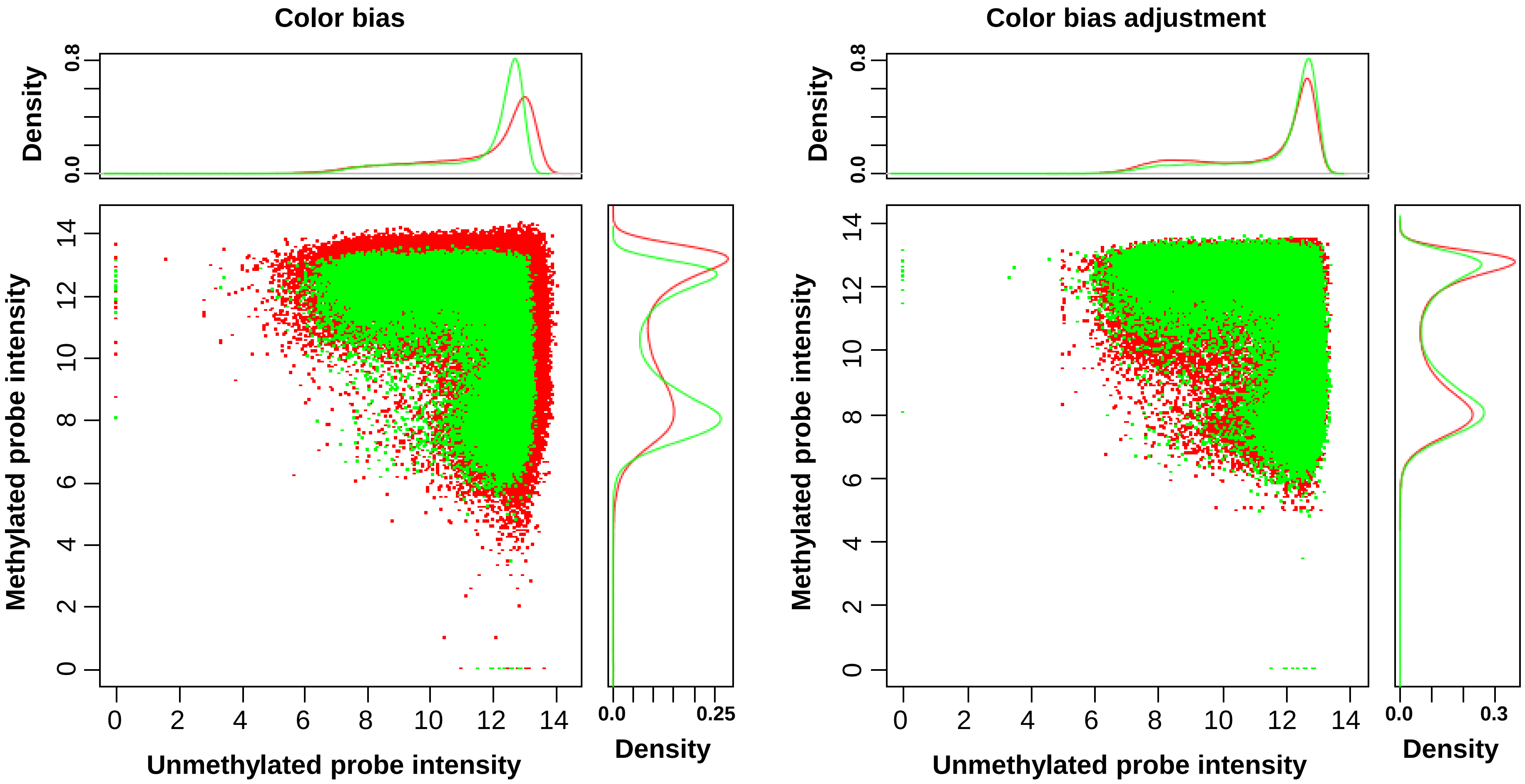
Color bias
Color bias adjustment
Density
0.0
0.8
Methylated probe intensity
0
2
4
6
8
10
12
14
Unmethylated probe intensity
0.25
0.3

Figure S1

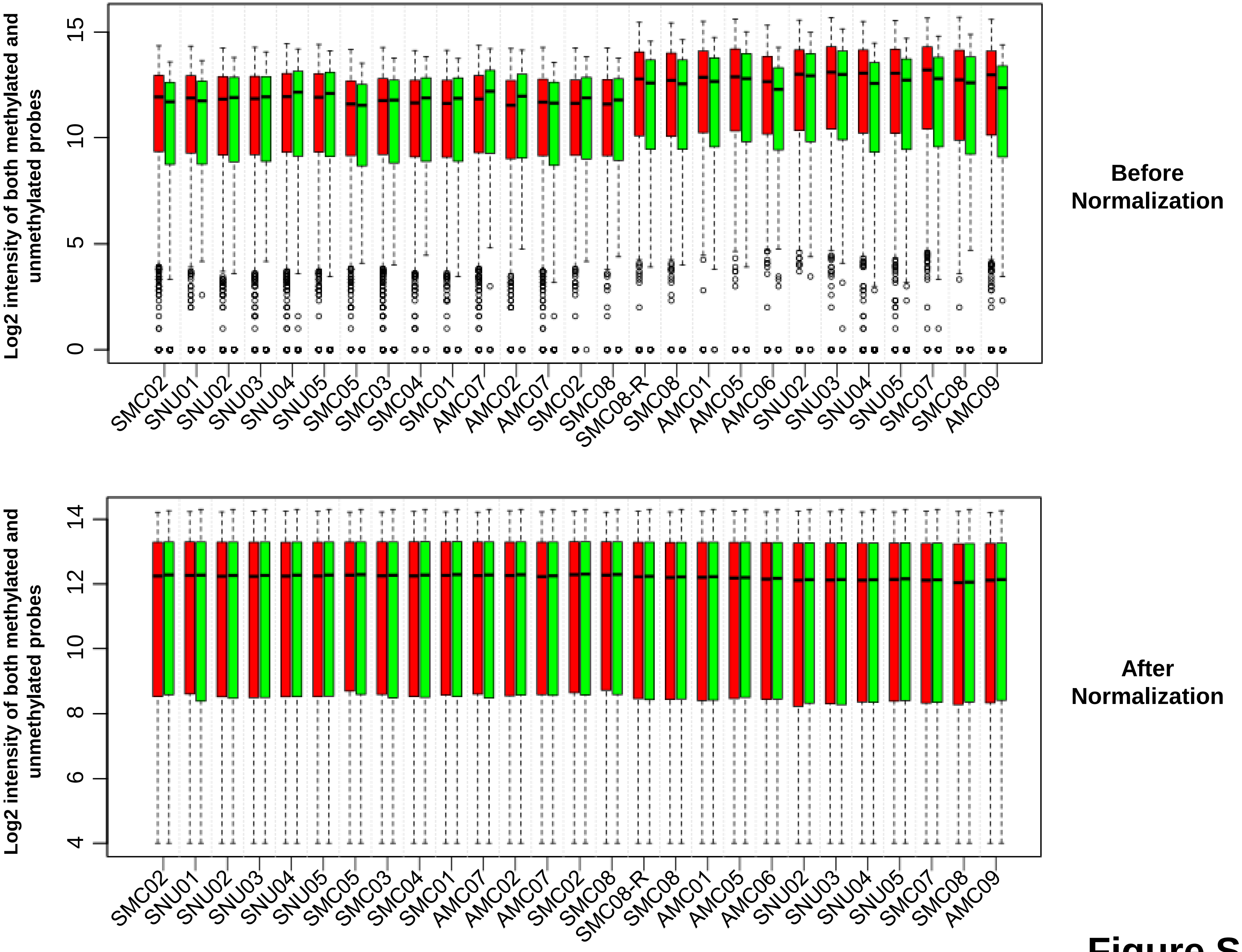

Log2 intensity of both methylated and unmethylated probes
15
10
5
0
SMC02 SNU01 SNU02 SNU03 SNU04 SNU05 SMC05 SMC03 SMC04 SMC01 AMC07 AMC02 AMC07 SMC02 SMC08 SMC08-R SMC08 AMC01 AMC05 AMC06 SNU02 SNU03 SNU04 SNU05 SMC07 SMC08 AMC09
Before Normalization
Log2 intensity of both methylated and unmethylated probes
14
12
10
8
6
4
SMC02 SNU01 SNU02 SNU03 SNU04 SNU05 SMC05 SMC03 SMC04 SMC01 AMC07 AMC02 AMC07 SMC02 SMC08 SMC08-R SMC08 AMC01 AMC05 AMC06 SNU02 SNU03 SNU04 SNU05 SMC07 SMC08 AMC09
After Normalization

Figure S2

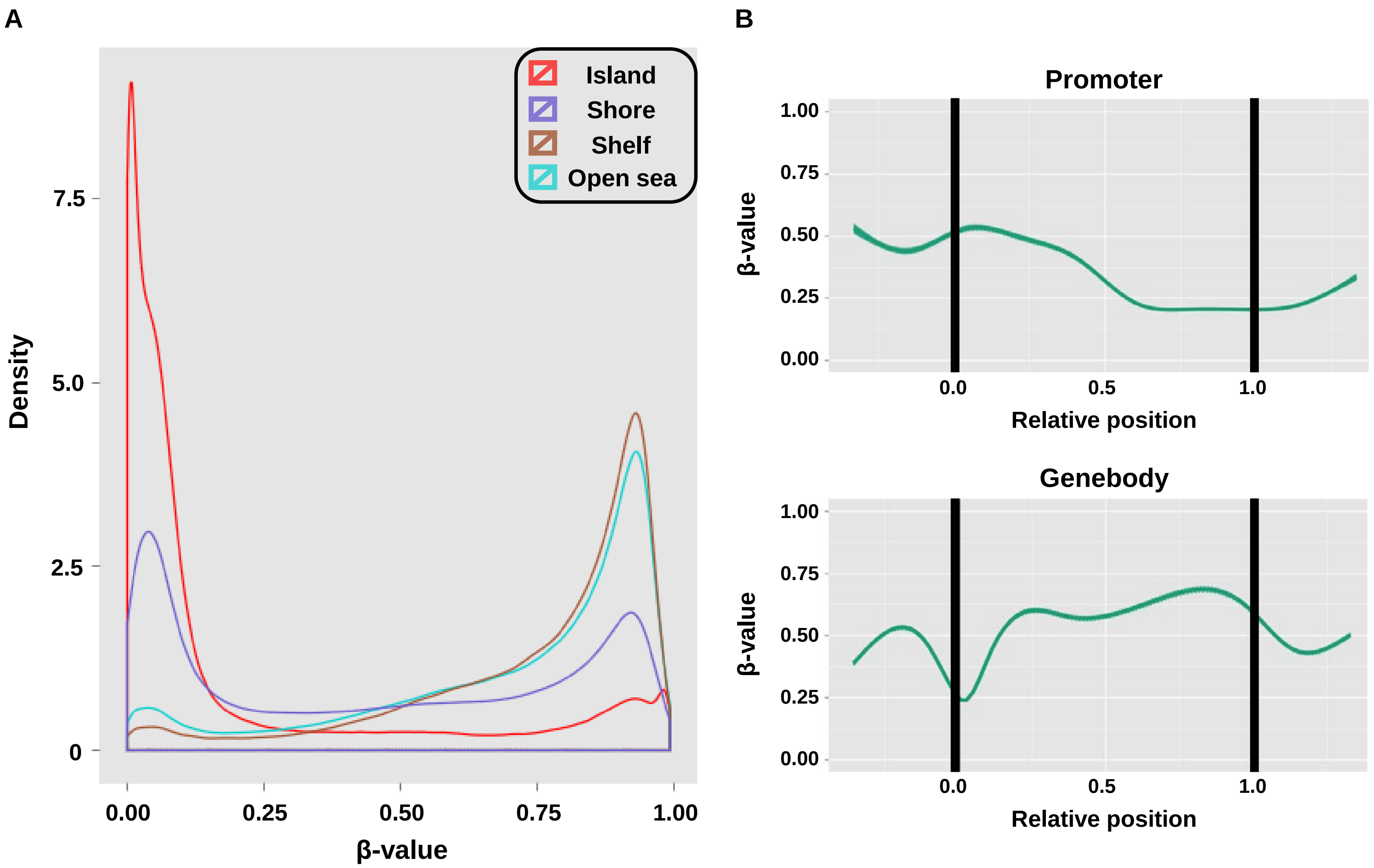
A
Island
Shore
Shelf
Open sea
Density
7.5
5.0
2.5
0
0.00
0.25
0.50
0.75
1.00
β-value
B
Promoter
1.00
0.75
0.50
0.25
0.00
β-value
0.0
0.5
1.0
Relative position
Genebody
1.00
0.75
0.50
0.25
0.00
β-value
0.0
0.5
1.0
Relative position

Figure S3

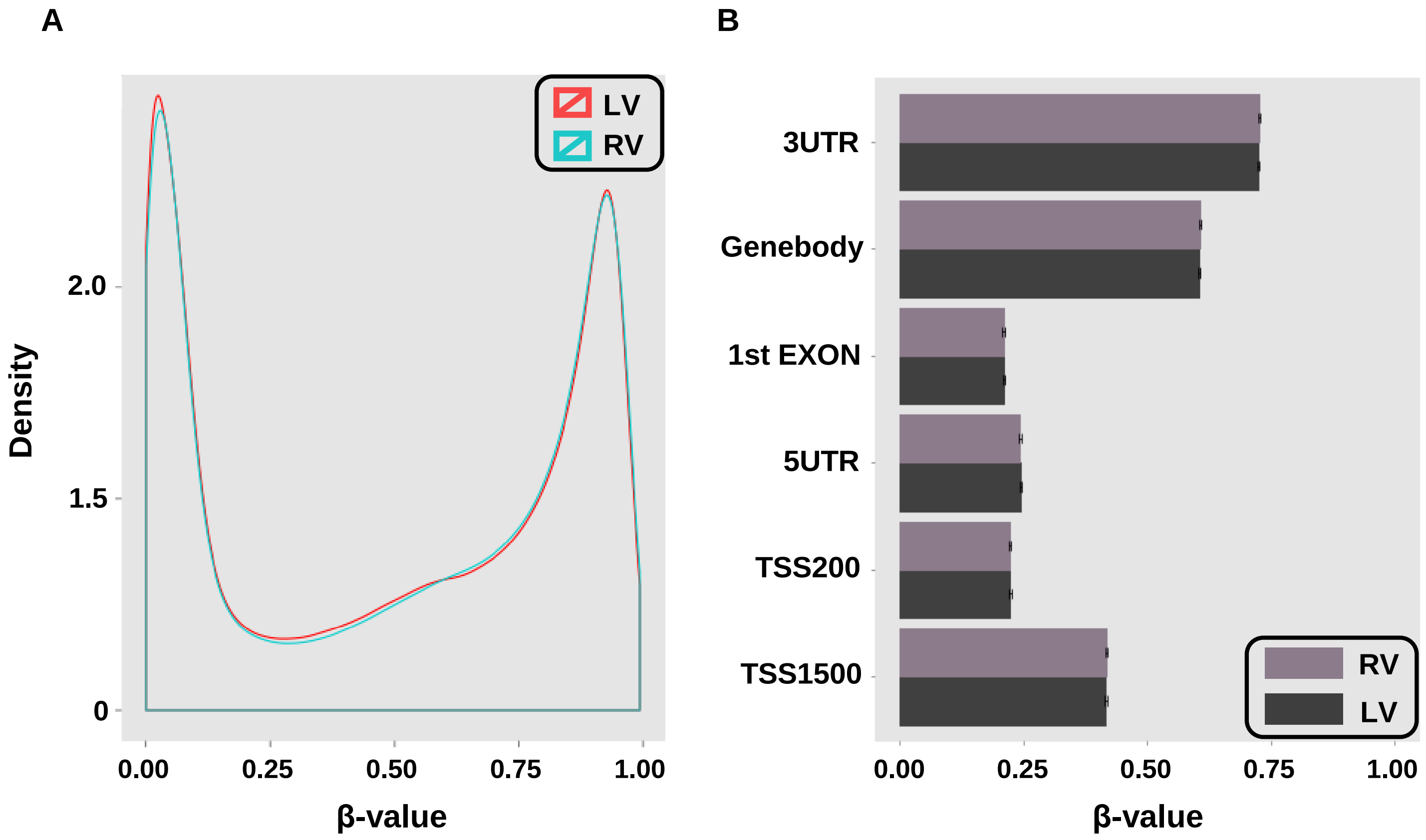
A
B
LV
RV
Density
2.0
1.5
0
0.00
0.25
0.50
0.75
1.00
β-value
3UTR
Genebody
1st EXON
5UTR
TSS200
TSS1500
RV
LV
0.00
0.25
0.50
0.75
1.00
β-value

Figure S4

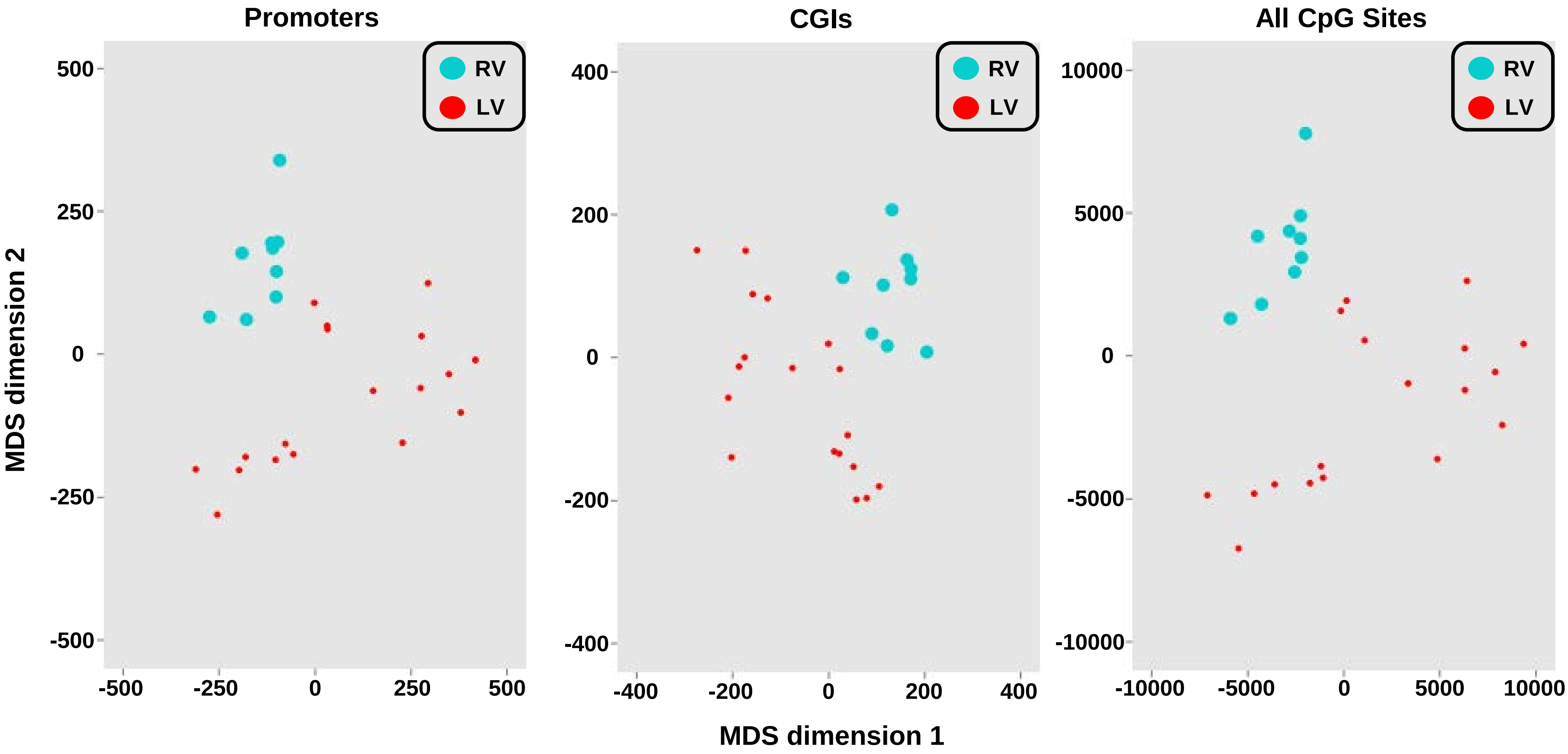

Promoters
CGIs
All CpG Sites
RV
LV
RV
LV
RV
LV
MDS dimension 2
MDS dimension 1
500
250
0
-250
-500
-500
-250
0
250
500
400
200
0
-200
-400
-400
-200
0
200
400
10000
5000
0
-5000
-10000
-10000
-5000
0
5000
10000

Figure S5

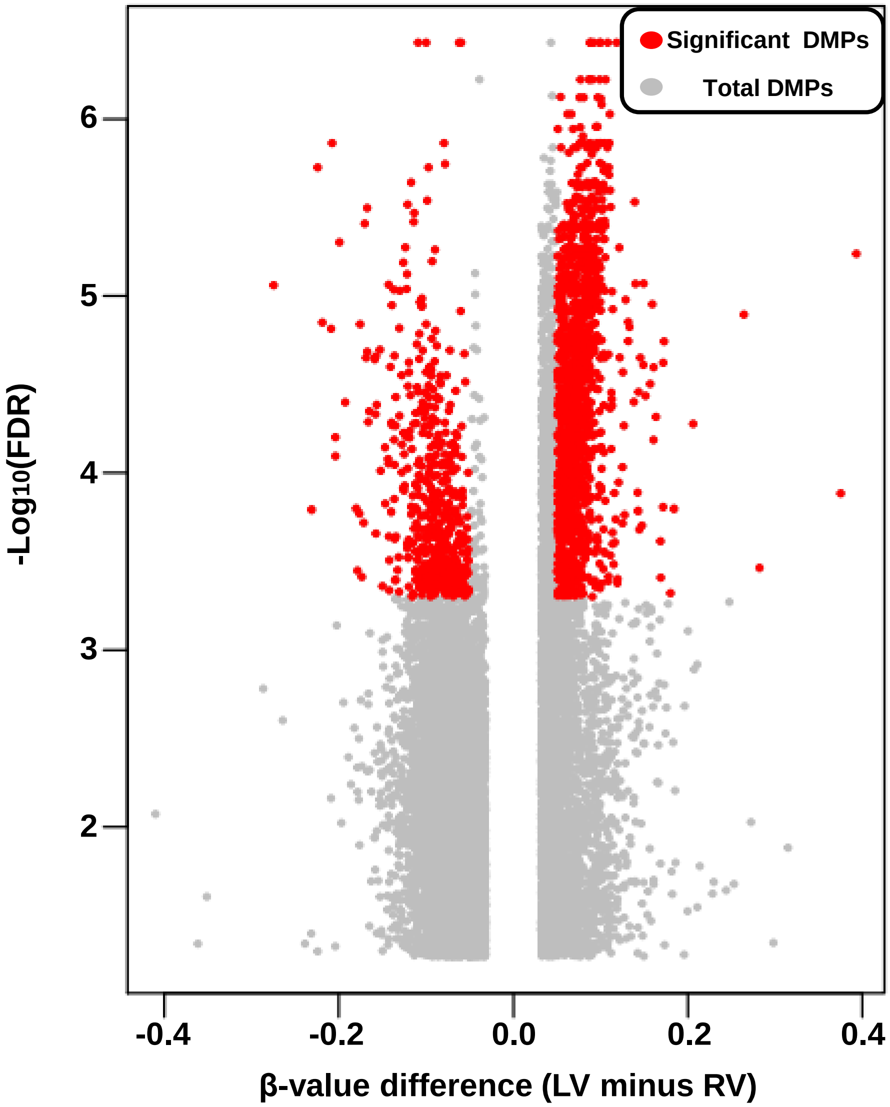
Significant DMPs
Total DMPs
-Log10(FDR)
6
5
4
3
2
-0.4
-0.2
0.0
0.2
0.4
β-value difference (LV minus RV)

Figure S6

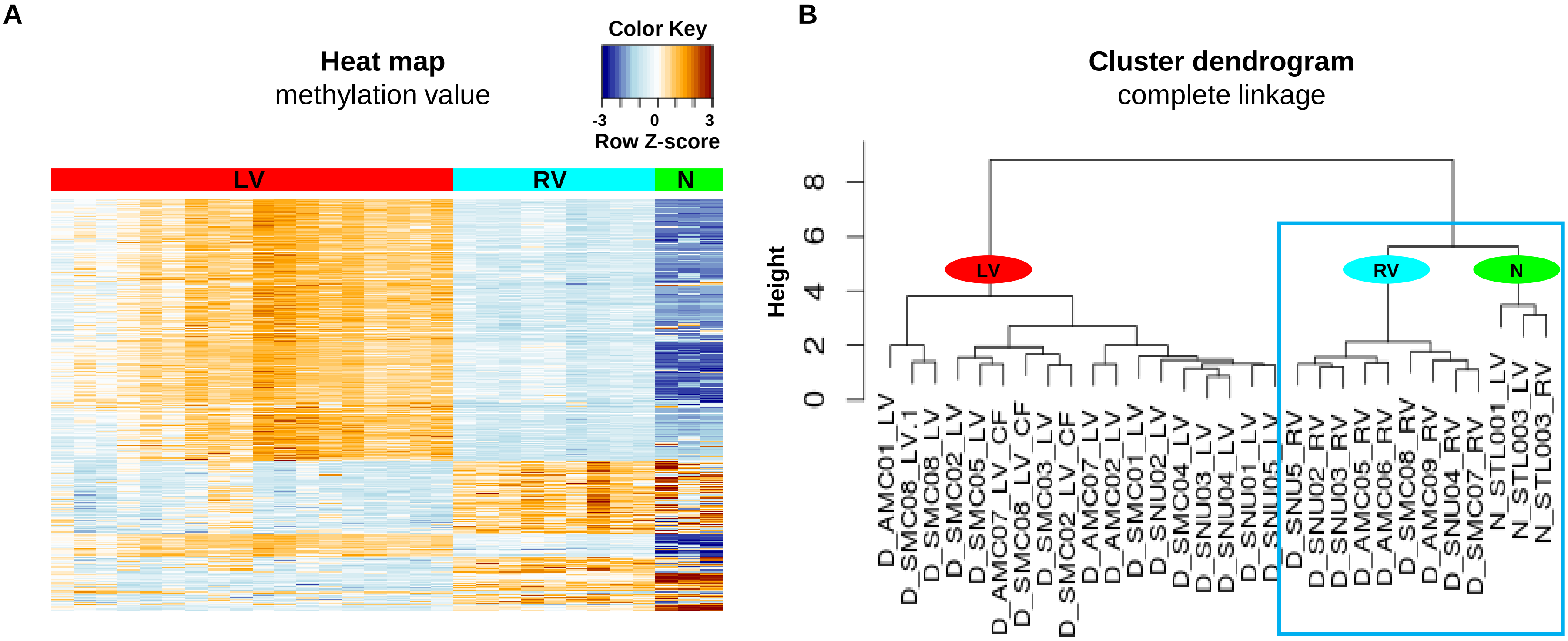
A
Heat map
methylation value
Color Key
-3
0
3
Row Z-score
LV
RV
N
B
Cluster dendrogram
complete linkage
Height
0
2
4
6
8
LV
RV
N
D_AMC01_LV
D_SMC08_LV.1
D_SMC08_LV
D_SMC02_LV
D_SMC05_LV
D_AMC07_LV_CF
D_SMC08_LV_CF
D_SMC03_LV
D_SMC02_LV_CF
D_AMC07_LV
D_AMC02_LV
D_SMC01_LV
D_SNU02_LV
D_SMC04_LV
D_SNU03_LV
D_SNU04_LV
D_SNU01_LV
D_SNU05_LV
D_SNU5_RV
D_SNU02_RV
D_SNU03_RV
D_AMC05_RV
D_AMC06_RV
D_SMC08_RV
D_AMC09_RV
D_SNU04_RV
D_SMC07_RV
N_STL001_LV
N_STL003_LV
N_STL003_RV

Figure S7

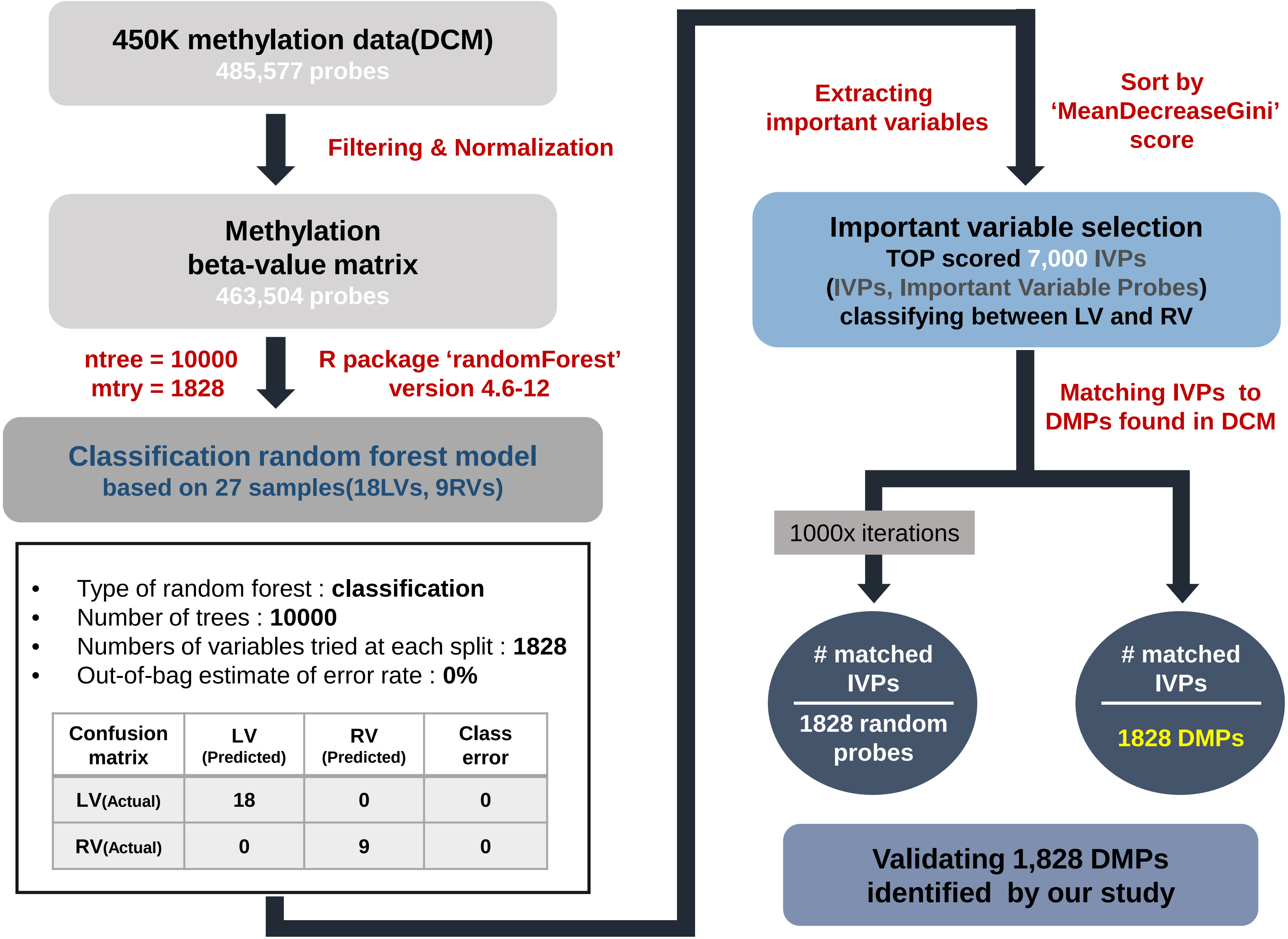

Confusion matrix	LV (Predicted)	RV (Predicted)	Class error
LV(Actual)	18	0	0
RV(Actual)	0	9	0

Figure S8

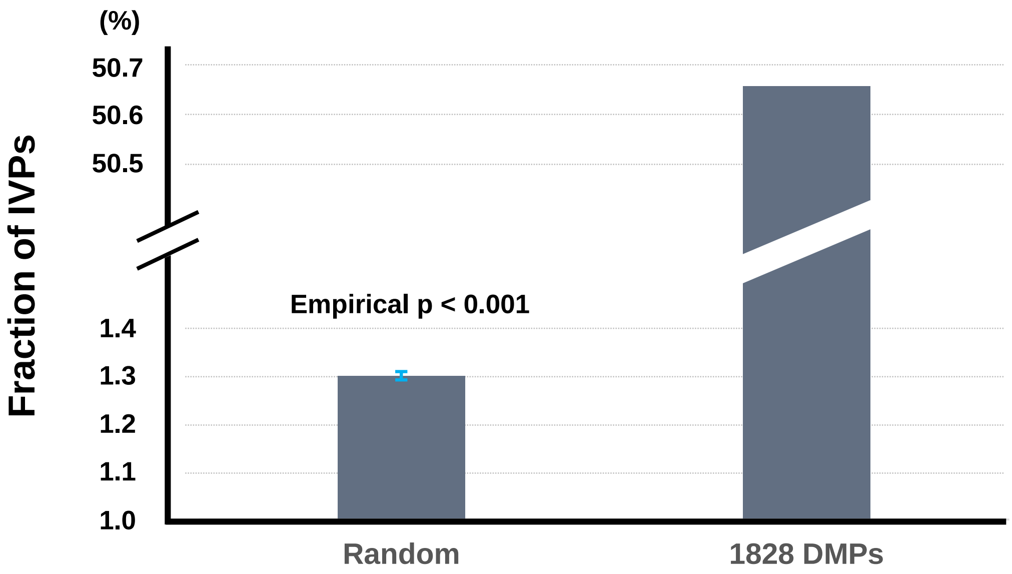

(%)
50.7
50.6
50.5
1.4
1.3
1.2
1.1
1.0
Fraction of IVPs
Empirical p < 0.001
Random
1828 DMPs

Figure S9

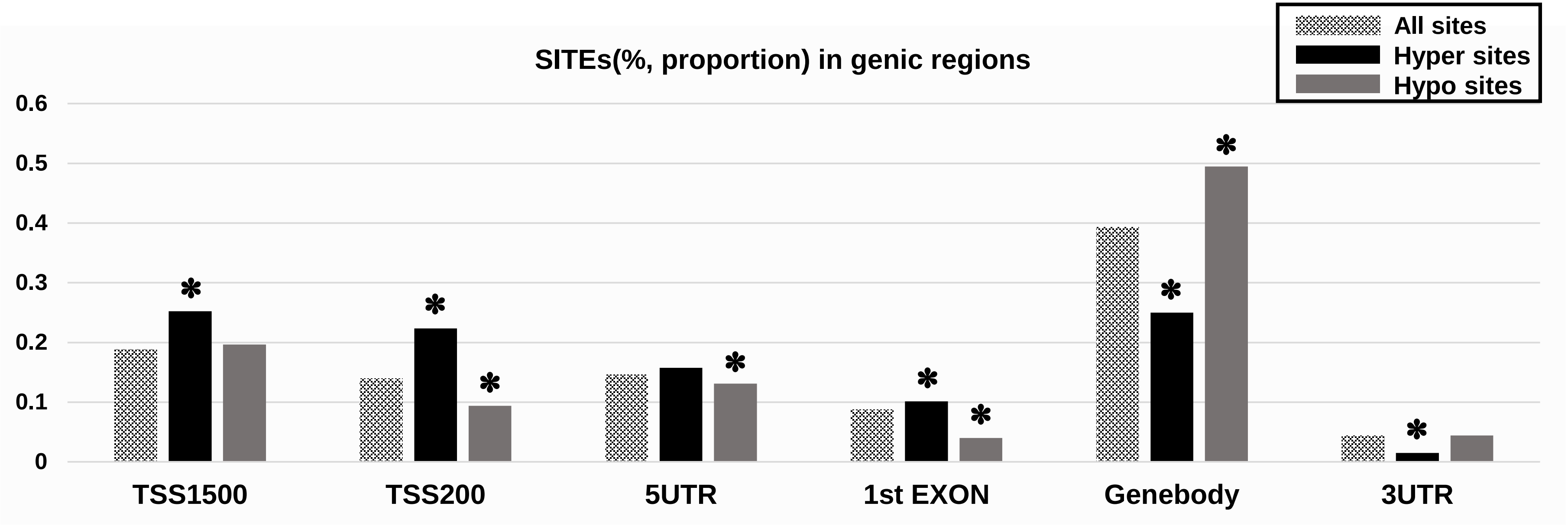
SITEs(%, proportion) in genic regions
All sites
Hyper sites
Hypo sites
0.6
0.5
0.4
0.3
0.2
0.1
0
TSS1500
TSS200
5UTR
1st EXON
Genebody
3UTR

Figure S10

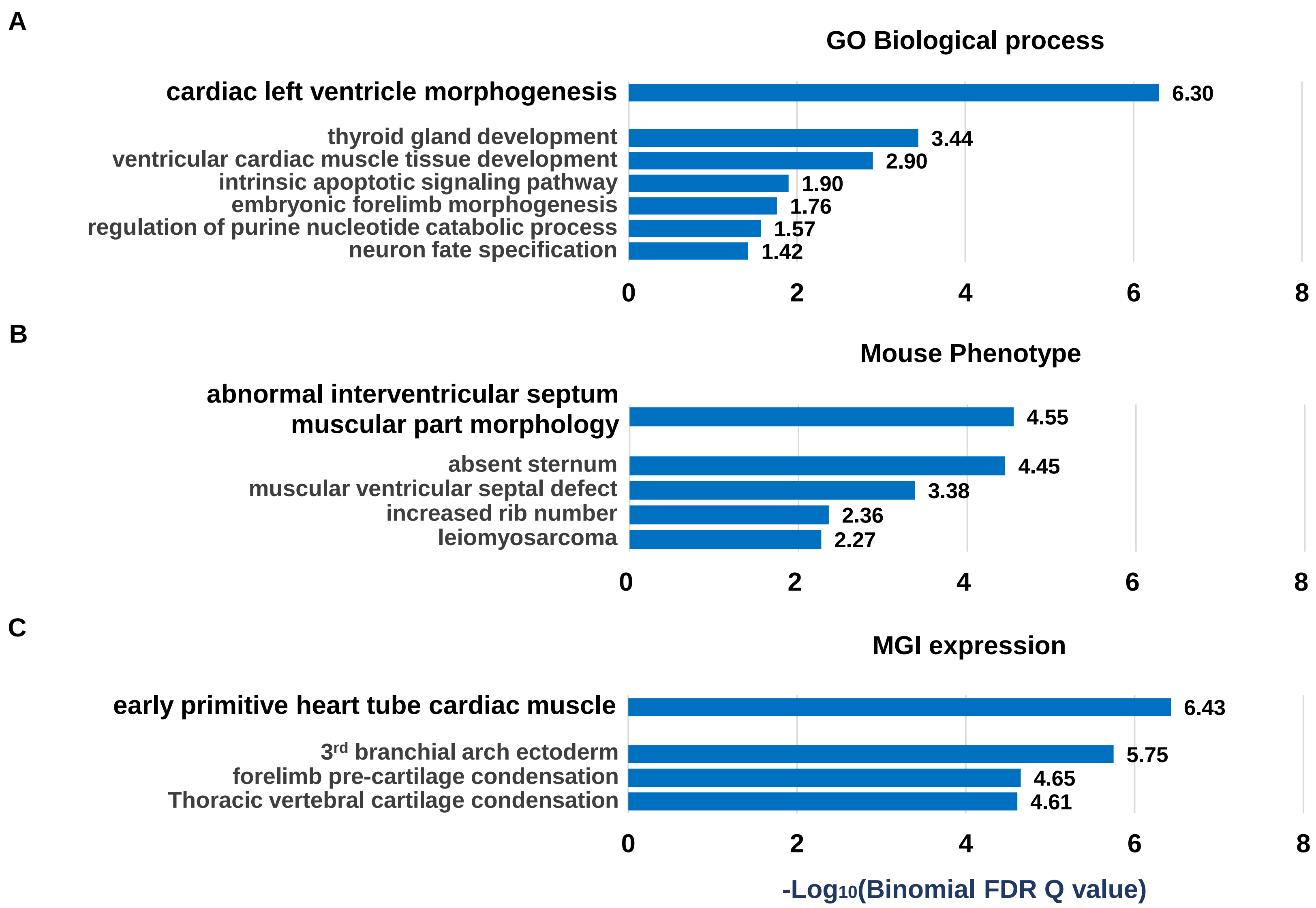

A
GO Biological process
cardiac left ventricle morphogenesis
thyroid gland development
ventricular cardiac muscle tissue development
intrinsic apoptotic signaling pathway
embryonic forelimb morphogenesis
regulation of purine nucleotide catabolic process
neuron fate specification
6.30
3.44
2.90
1.90
1.76
1.57
1.42
0 2 4 6 8
B
Mouse Phenotype
abnormal interventricular septum muscular part morphology
absent sternum
muscular ventricular septal defect
increased rib number
leiomyosarcoma
4.55
4.45
3.38
2.36
2.27
0 2 4 6 8
C
MGI expression
early primitive heart tube cardiac muscle
3rd branchial arch ectoderm
forelimb pre-cartilage condensation
Thoracic vertebral cartilage condensation
6.43
5.75
4.65
4.61
0 2 4 6 8
-Log10(Binomial FDR Q value)

Figure S11

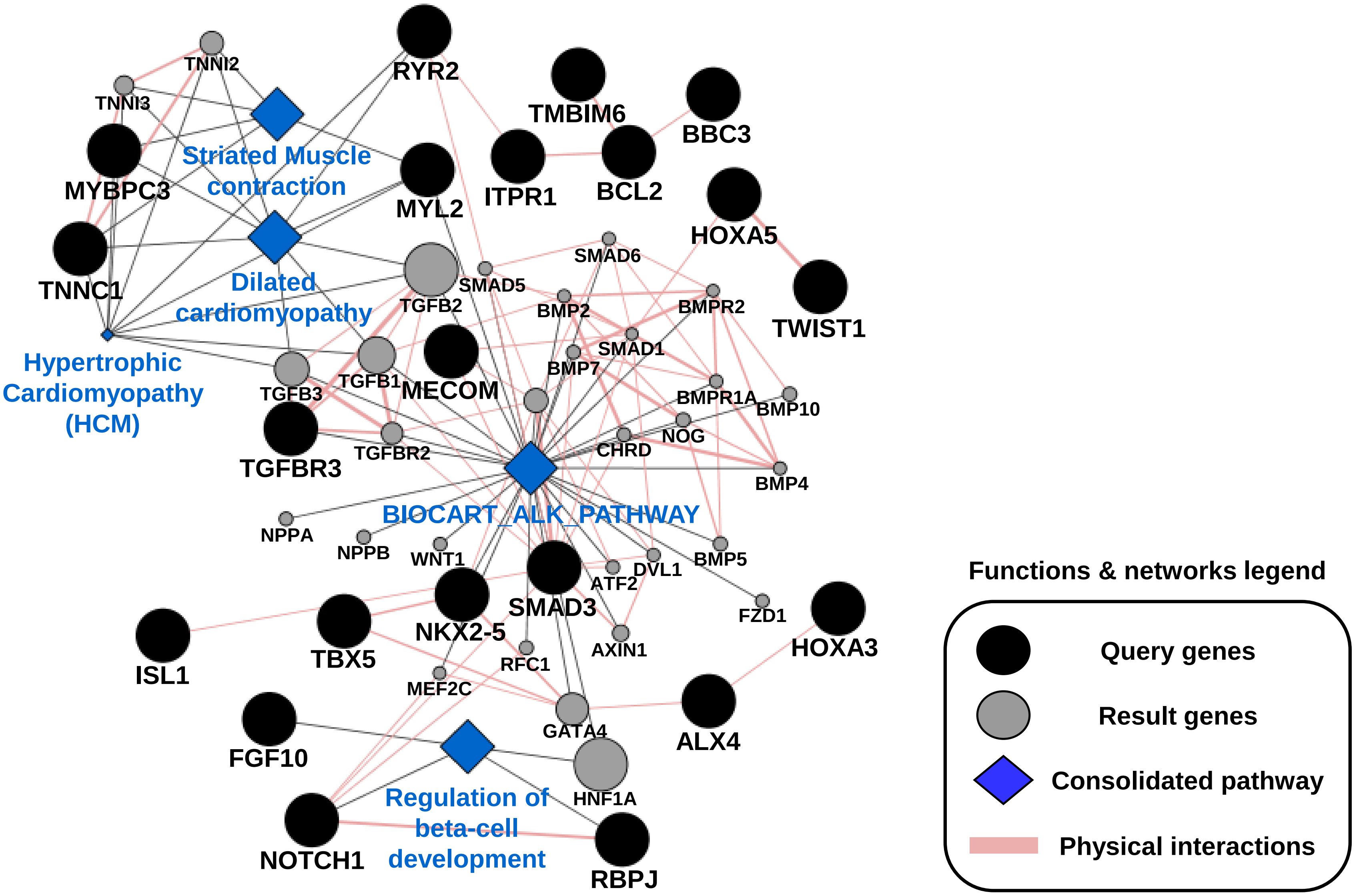
TNNI2
TNNI3
RYR2
TMBIM6
BBC3
Striated Muscle contraction
MYBPC3
MYL2
ITPR1
BCL2
HOXA5
TNNC1
Dilated cardiomyopathy
SMAD6
SMAD5
TGFB2
BMP2
BMPR2
TWIST1
SMAD1
Hypertrophic Cardiomyopathy (HCM)
BMP7
TGFB3
TGFB1
MECOM
BMPR1A
BMP10
NOG
CHRD
TGFBR2
TGFBR3
BMP4
BIOCART_ALK_PATHWAY
NPPA
NPPB
WNT1
BMP5
DVL1
ATF2
SMAD3
NKX2-5
FZD1
HOXA3
ISL1
TBX5
RFC1
AXIN1
MEF2C
GATA4
ALX4
FGF10
HNF1A
Regulation of beta-cell development
NOTCH1
RBPJ
Functions & networks legend
Query genes
Result genes
Consolidated pathway
Physical interactions

Figure S12

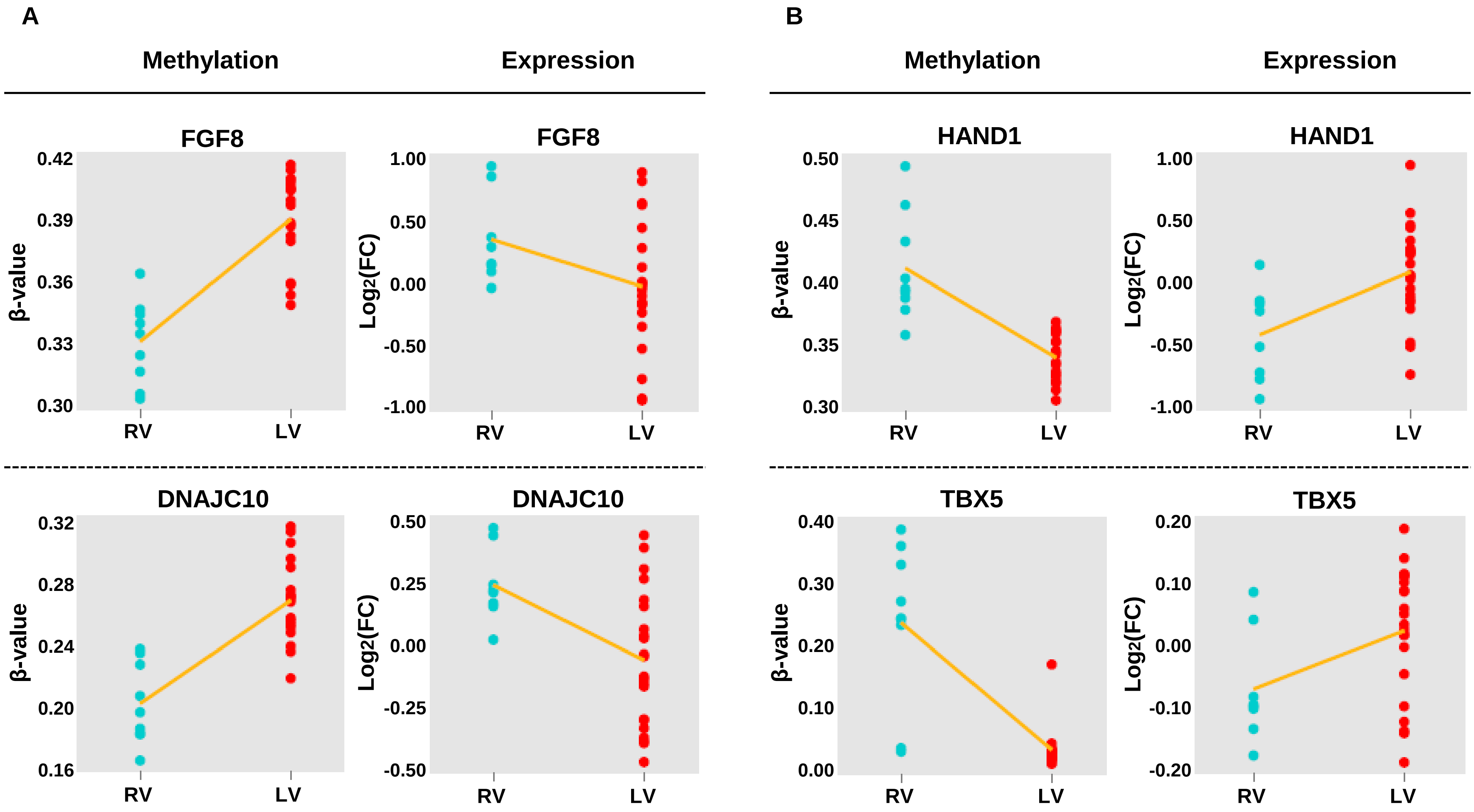
A
Methylation
Expression
FGF8
β-value
0.42
0.39
0.36
0.33
0.30
RV
LV
FGF8
Log2(FC)
1.00
0.50
0.00
-0.50
-1.00
RV
LV
DNAJC10
β-value
0.32
0.28
0.24
0.20
0.16
RV
LV
DNAJC10
Log2(FC)
0.50
0.25
0.00
-0.25
-0.50
RV
LV
B
Methylation
Expression
HAND1
β-value
0.50
0.45
0.40
0.35
0.30
RV
LV
HAND1
Log2(FC)
1.00
0.50
0.00
-0.50
-1.00
RV
LV
TBX5
β-value
0.40
0.30
0.20
0.10
0.00
RV
LV
TBX5
Log2(FC)
0.20
0.10
0.00
-0.10
-0.20
RV
LV

Figure S13
